# Extrapulmonary *Mycobacterium abscessus* Infections, France, 2012–2020^1^

**DOI:** 10.3201/eid3011.240459

**Published:** 2024-11

**Authors:** Benoît Heid-Picard, Faiza Mougari, Anne Pouvaret, Fanny Lanternier, Zeina Awad, Emmanuelle Bille, Olivier Lortholary, Emmanuelle Cambau, the Mabsc Study Group

**Affiliations:** Université Paris Cité, Paris, France

**Keywords:** nontuberculous mycobacteria, tuberculosis and other mycobacteria, Mycobacterium abscessus, emerging communicable diseases, healthcare-associated infection, travel medicine, extrapulmonary, France

## Abstract

*Mycobacterium abscessus* infection is challenging to treat. Extrapulmonary *M.* *abscessus* infections (EP-MAB) are less common than pulmonary *M.* *abscessus* infections. To evaluate treatment regimens, we retrospectively analyzed consecutive microbiologically confirmed EP-MAB cases diagnosed in France during 2012–2020. We studied 45 patients with EP-MAB, including 14 bone and joint infections, 10 skin and soft tissue infections, and 8 lymph node infections. Most (62%) patients had no reported immunodeficiency. In 27 patients, EP-MAB followed healthcare-associated (44%) or environmental (16%) injuries. Of the 45 isolates, 25 were subspecies *abscessus*, 10 *bolletii*, and 9 *massiliense*; 1 was unidentified. Cure was achieved for 36 (80%) patients who received a median antimicrobial regimen of 6 months; 22 (55%) also underwent surgery. Four patients died, and 5 were unavailable for follow-up. EP-MAB predominantly affects immunocompetent patients after an injury; outcomes are favorable. We propose a >6-month regimen of antimicrobial therapy with consideration for surgery and regular patient reassessment.

Nontuberculous mycobacteria (NTM) are found in the environment (*1*,*2*). They are increasingly recognized as causative agents of infections regardless of patient age or immune status, and in several countries they surpass tuberculosis in terms of prevalence (*3*–*5*).

*Mycobacterium abscessus* is a rapidly growing NTM (*6*,*7*), subcategorized as 3 subspecies: *abscessus*, *massiliense*, and *bolletii*. Subspecies affect pulmonary infection outcomes (*8*) because they correlate with the expression of the *erm*(41) gene (*9*), conferring inducible resistance to macrolides. Resistance has been noted for *M. abscessus* subsp. *bolletii* and *M. abscessus* subsp. *abscessus* sequevar T28 isolates, and susceptibility has been noted for *M. abscessus* subsp. *massiliense* and *M. abscessus* subsp. *abscessus* sequevar C28 isolates (*10*). Infection with those subspecies contributes to a poorer clinical outcome, restricting the effectiveness of macrolides, although they are recommended for pulmonary infections for all *M. abscessus* strains (*10*). 

*M. abscessus* primarily causes pulmonary infections, particularly in patients with bronchiectasis (*10*). Despite multidrug antimicrobial therapy, cure rates remain <50%, and mortality rates are high (*11*). Extrapulmonary *M. abscessus* infections (EP-MAB) are rare, documented through localized outbreaks in single centers (*12*–*17*) or case series (*18*).

In France, *M. abscessus* accounts for 20%–25% of NTM clinical isolates received by the French National Reference Centre for Mycobacteria (CNR-MyRMA; https://cnrmyrma.fr) for identification and antimicrobial susceptibility testing in an infection context (E. Cambau, unpub. data). Although previous guidelines (*19*) provided advice about extrapulmonary NTM infections and proposed macrolide-based antimicrobial regimens and surgery and new guidelines have recently been updated for pulmonary *M. abscessus* infections (*10*), there are no specific recommendations for EP-MAB treatment duration. To investigate EP-MAB medical management (e.g., diagnostis, treatments, and outcomes), we retrospectively studied consecutive patients with microbiologically confirmed EP-MAB. In accordance with French law, our study protocol received approval by the “Comité d’éthique de la recherche AP-HP Centre” (IRB registration no. 00011928, Réf. 2020–12–04).

## Material and Methods

### Case Eligibility Criteria

We reviewed cases involving extrapulmonary infections associated with *M. abscessus* strains among all strains registered in the CNR-MyRMA database during January 2012–April 2020. Cases were eligible if they met the criteria of clinical signs/symptoms dependent on the site of the infection and >1 *M. abscessus* isolate was concurrently recovered from a sample from an extrapulmonary site (e.g., skin biopsy sample, articular fluid, blood). Multisite infections were defined as those involving 2 nonadjacent organs with concordant clinical, microbiological, or histologic criteria. Multisite infections that included pulmonary localization were eligible, and cases of pulmonary infection alone were not. The inclusion was assessed by authors B.H.P. and A.P.

### Data Retrieval

We sent a questionnaire gathering epidemiologic, clinical, biological, radiologic, therapeutic, and follow-up data to physicians and microbiologists who reported a case. When needed, we also contacted them by phone or mail to request medical reports.

For injury-related infections, we set the geographic origin as the location where the injury occurred. For infections not associated with injury, the geographic origin was the location of the patient when initial symptoms were noted and, if that information was unavailable, the location where the diagnostic specimen was obtained. Healthcare-associated infections were defined as those following medical, surgical, or aesthetic procedures performed before the infection and at the same location. Antimicrobial regimens were recorded if administered for >7 days. We presented qualitative variables as medians (ranges). We considered patients cured if clinical reports indicated such; no microbiological evidence was required.

### Identification and Antibiotic Susceptibility

We conducted mycobacterial identification and antimicrobial susceptibility testing (AST) in accordance with standard practice (*10*,*20*) and strain identification by using the GenoType *Mycobacterium* CM kit (Hain Lifescience, https://www.hain-lifescience.de), IVD MALDI Biotyper (Brucker Daltronics, https://www.bruker.com), and mass spectrometry Microflex LT MALDI-TOF MS (Brucker Daltronics), analyzed with the Mycobacteria Library MBT Compass3, (Bruker Daltonics) subspecies. We determined *erm*(41) sequevars by using the Genotype NTM-DR test (Hain Lifesciences, Bruker Daltronics) (*21*) complemented with *hsp65* or *rpoB* PCR Sanger sequencing when necessary. We performed AST in calcium-supplemented Mueller-Hinton medium and used Sensititre Myco AST Plate (Thermo Scientific, https://www.thermofisher.com) to determine MICs (*10*,*22*). To detect inducible clarithromycin resistance, we determined clarithromycin MICs after 3–5 days and after 14 days of incubation (*9*,*23*). We conducted molecular detection of mutations associated with antimicrobial resistance by targeting *rrl* for macrolides and *rrs* for aminoglycosides (*24*). We identified the *erm*(41) sequevar through PCR sequencing (*24*).

## Results

**Figure 1:**
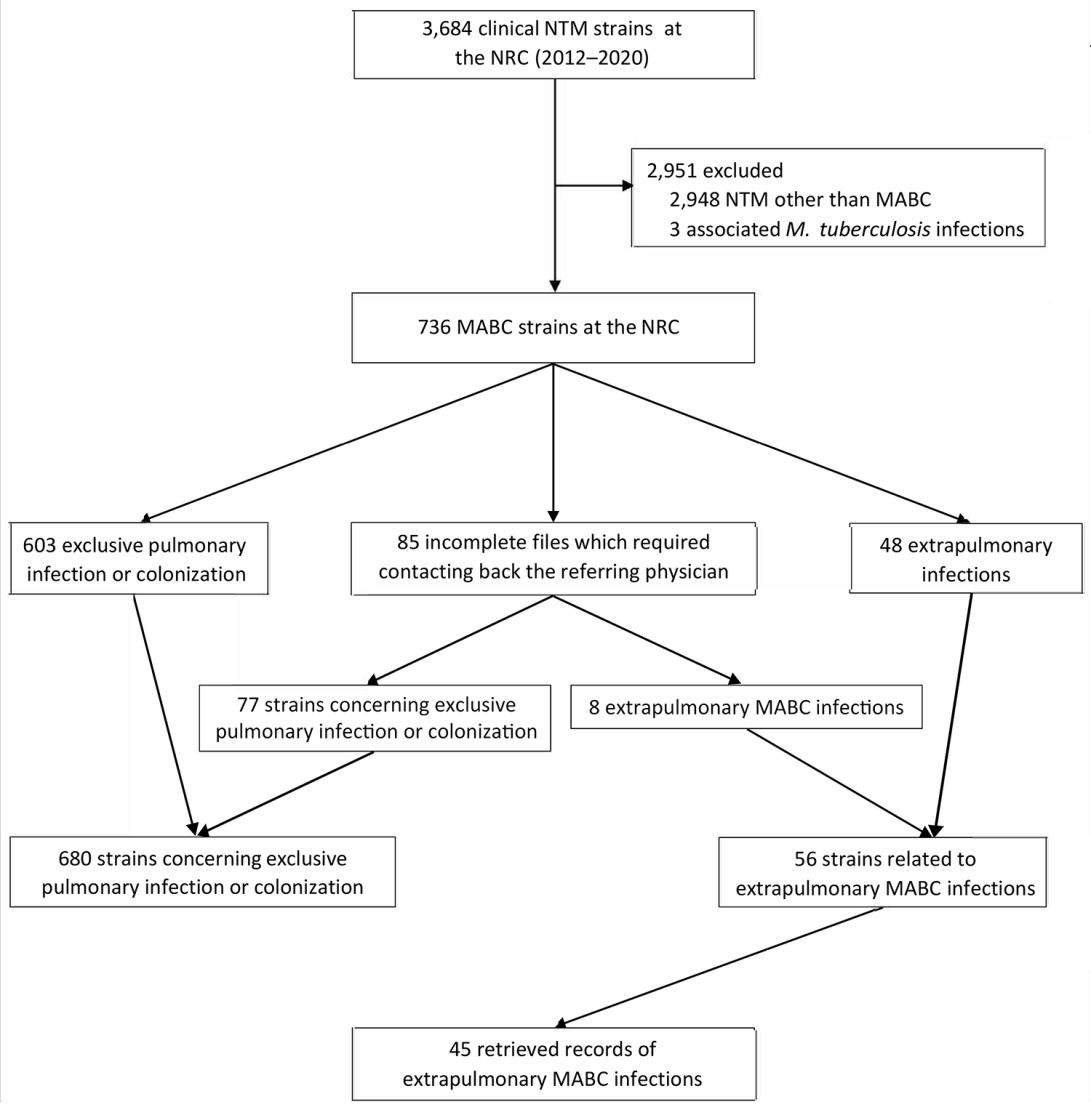
Flowchart of selection for study of extrapulmonary *Mycobacterium abscessus* infections, France, 2012–2020. NRC, national reference center; NTM, nontuberculous mycobacteria; MABC, *Mycobacterium abscessus* complex.

During 2012–2020, CNR-MyRMA received 3,684 NTM strains for identification, including 736 (20%) *M. abscessus* strains, among which 56 (8%) were associated with EP-MAB (Figure 1). The average annual ratio of extrapulmonary to pulmonary strains was 0.07 (range 0.02–0.15) (Appendix Figure). Clinical data were collected for 47 (84%) strains isolated from the 45 patients (Table; Appendix Table 1).

**Table:** Characteristics of 45 extrapulmonary *Mycobacterium abscessus* infections, France, 2012–2020*

Variable	Infections			Total
	Bone/joint	Skin/soft tissue	Lymph node	
Patients, no. (%)	14 (30)	10 (22)	8 (18)	45 (100)
Mean age, y, ± SD	50.2 ± 26.6	60.8 ± 21.2	43.7 ± 25.3	51.4 ± 24.5
M/F sex ratio	1.33	0.66	1	0.88
Geographic origin, no. (%)				
Metropolitan France	3	4	2	13 (29)
Overseas French territories	4	3	2	13 (29)
Foreign countries	7	3	4	19 (42)
Underlying immune disorder, no. (%)				
No known immunodeficiency	13	4	5	28 (62)
Immunosuppressive treatment	1	5	2	9 (20)
Solid tumor	0	0	1	4 (9)
Malignant hemopathy	0	1	0	2 (4)
Clinical features, no. (%)				
B symptoms†	7	4	2	17 (38)
Skin involvement	4	10	2	22 (49)
Initial injury	12	3	3	27 (60)
Median months to diagnosis [range]	4.5 [0.25–72]	1.5 [1–72]	1 [0.25–3]	3 [0.25–72]
Environmental	4	1	2	7 (16)
Healthcare associated	8	2	1	20 (44)
*M. abscessus* subspecies and *erm*(41) sequevars, no. (%)				
*abscessus* T28	7	5	1	19 (40)
*abscessus* C28	2	1	0	6 (13)
* bolletii*	2	2	4	12 (26)
* massiliense*	3	2	2	9 (19)
Medical management, no. (%)				
Surgery + antimicrobial regimen	11	3	3	22 (49)
Surgery alone	1	0	1	3 (7)
Antimicrobial regimen alone	2	6	3	13 (29)
Media months of antimicrobial regimen duration [range]	6 [2–12]	6 [3–15]	6 [1–6]	6 [1–long-term]
Outcome, no. (%)				
Cure	13	7	7	33 (73)
Relapse then cured	0	0	0	3 (7)
Not available for follow-up	1	2	1	5 (11)
Death	0	1	0	4 (9)

*Values are no. (%) except as indicated. †Fever, night sweats, or weight loss.

### Clinical and Biological Features

The 45 cases were distributed as 14 (31%) bone and joint infections (BJIs), 10 (22%) skin and soft tissue infections (SSTIs), 8 (18%) lymph node infections (LNIs), 4 (9%) bacteremia or catheter-related infections, 4 (9%) multisite infections, 3 (7%) breast infections, and 2 (4%) biliary tract infections. The median time between initial signs/symptoms and diagnosis was 2 months (range 1 week–2 years); 89% of cases were diagnosed in <6 months. The clinical signs were cutaneous lesions in 22 (46%) patients and palpable lymph nodes in 6 (13%) patients. Fever was noted for 9 (20%) patients, asthenia for 8 (17%), and night sweats for 2 (4%). BJIs were 7 monoarthritis, 4 lower limb osteitis, 2 spondylodiscitis, and 1 hip arthroplasty. Few details were available for SSTI descriptions; 7 cases were reported as cutaneous nodules. LNIs involved cervical lymph nodes in 4 patients, mediastinal lymph nodes in 3, and inguinal lymph nodes in 1. Median neutrophil count was 4.1 × 10^9^ cells/L (range 0.93–11.64 × 10^9^ cells/L), median lymphocyte count was 1.2 × 10^9^ cells/L (range 0.1–3.3 × 10^9^ cells/L), and median monocyte count was 0.6 × 10^9^ cells/L (range 0.08–1.77 × 10^9^ cells/L). Median C-reactive protein concentration was 29 mg/L (range <5–230 mg/L). Thoracic computed tomography was available for 21 (47%) patients and revealed parenchymal infiltrates (including mostly condensations, nodules, or both) in 15 patients. After other diseases were excluded, pulmonary involvement was confirmed for only 2 of the 15 patients. For 2 of 4 patients with multisite *M. abscessus* infection, a thoracic computed tomography scan showed mediastinal lymph nodes in one and bilateral pulmonary nodules in the other. For the 2 patients with microbiologically proven spondylodiscitis, respiratory samples were also *M. abscessus* despite the absence of pulmonary signs or known underlying bronchopulmonary disease, suggesting probable colonization.

### Epidemiology of Patients with Extrapulmonary Infections

**Figure 2:**
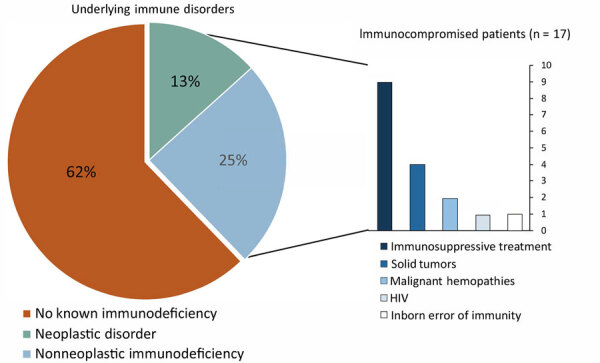
Medical conditions associated with extrapulmonary *M. abscessus* infections, France, 2012–2020.

The median patient age was 51.4 years (range 1–98 years); 6 (13%) were <18 years of age. The M:F sex ratio was 0.88. Underlying disease was documented for 19 (42%) patients, including 16 with immunodeficiency acquired by treatment or disease (Figure 2), 1 with inborn error of immunity (NEMO [nuclear factor κB essential modulator] mutation), and 2 with prior chronic pulmonary diseases. The remaining 26 patients were classified as immunocompetent, 2 of whom were negative for inborn immunodeficiency.

Penetrating injury was suspected as the portal of entry for 27 (60%) patients, including 20 (44%) with healthcare-related cases and 7 (16%) associated with an environmental source. The median time after injury was 3 months (range 1 week–5 years). Of the 14 BJIs, 11 (79%) followed an initial injury, which was either skin injury (all 4 osteitis) or articular infiltration (3 of 7 monoarthritis). The 3 breast infections were associated with healthcare injury, 2 after bilateral prosthetic implant procedures performed in Mauritius and 1 after breast biopsy. Of 4 patients who experienced bacteremia, 3 had an implantable venous access device. Of 10 SSTIs, 3 were associated with injury, 2 of them on tattoos.

Travel-related infection affected 32 (71%) patients, of which 13 (29%) of 45 cases were diagnosed in overseas France (6 in French West Indies, 4 in La Réunion Island, 1 in French Guyana, 1 in New Caledonia, and 1 in French Polynesia), 10 in Africa, 3 in the Americas, 3 in East Asia, 2 in Europe, and 1 in the Middle East. The remaining 13 (29%) patients were metropolitan France residents with no travel history that could be associated with *M. abscessus* infection.

### Microbiological Results

The 47 strains collected for the 45 cases were distributed as 25 (53%) *M. abscessus* subsp. *abscessus* that included 19 (40%) *erm*(41) sequevar T28 and 6 (13%) sequevar C28, 12 (26%) *M. abscessus* subsp*. bolletii*, and 9 (19%) *M. abscessus* subsp. *massiliense* (Appendix Table 2). Subspecies identification was missing for 1 strain isolated in 2012. For the patient who experienced 2 relapses (patient 42), the same *M. abscessus* subsp*. bolletii* strain was isolated. All 9 strains of *M. abscessus* subsp. *massiliense* and 6 *M. abscessus* subsp. *abscessus erm*(41) sequevar C28 were susceptible to macrolides with a clarithromycin 90% MIC of 0.5 mg/L after 3–5 days and 1 mg/L after 14 days of incubation. For the 31 strains of *M. abscessus* subsp. *bolletii* and *M. abscessus* subsp. *abscessus erm*(41) sequevar T28, the clarithromycin 90% MIC was 8 mg/L after 3–5 days and >16 mg/L after 14 days of incubation. Sequences of *rrl* and *rrs* were available for all identified strains, and both were exhibited as a wild-type genotype.

### Treatment

Before diagnosis, none of the patients had been treated with a drug that targeted *M. abscessus*. Antimycobacterial agents were administered to 35 (77%) patients, including 22 (49%) who also underwent local surgery or local care; 3 (7%) patients underwent local surgery without antimicrobial drugs; 3 (7%) received local care alone; and 4 (9%) received no treatment (Appendix). Of the 35 initial antimicrobial drug regimens, 26 (74%) involved a combination of >3 drugs, 8 a combination of 2 drugs, and 1 was monotherapy. All regimens included macrolides for >3 weeks: clarithromycin (21 [62%]), azithromycin (11 [32%]), or sequential treatment with both (2 [6%]). For 30 (86%) patients, treatment started with an induction phase including >1 intravenous antimicrobial drug, and 4 (12%) patients received oral antimicrobial drugs only. For patients with BJIs, SSTIs, and LNIs, the median duration of the antimicrobial regimen was 6 months and the median duration of the induction phase was 6 weeks.

### Outcomes

Cure rate with no relapse was 73% (33 patients), including 4 patients who received neither antimicrobial drugs nor surgery: 3 infections resolved with local care alone, and 1 patient with LNI experienced spontaneous resolution after 1 month. During the follow-up period, 4 (9%) patients experienced relapses (3 of whom eventually experienced cure and the other was not available for follow-up) and 4 (9%) patients died (3 died before receiving treatment for *M. abscessu*s infection). Of the 4 who died, 2 had *M. abscessu*s bacteremia and received supportive care for cancer, 1 had *M. abscessu*s multisite infection diagnosed with a concomitant JC virus infection, and 1 had an SSTI and died of limb ischemia at 6 months of treatment. The 4 deaths were unrelated to *M. abscessus* infection. Five (11%) patients were unavailable for follow-up after 1 month of treatment. Overall cure was observed for 36 (80%) patients; median duration of patient follow-up was 32.5 months (1st quartile 26.25–3rd quartile 45.0 months). No explanatory factor was associated with the few infections that led to a negative outcome.

Immune reconstitution syndrome was noted for 1 patient (patient 25). The patient had an SSTI of the lower limb, and immune reconstitution syndrome affected the draining abdominal lymph nodes. The patient’s condition improved after administration of steroids.

## Discussion

*M. abscessus* is a challenging-to-treat pathogen (*10*), and EP-MAB is rarely described. In our series, EP-MAB often affected immunocompetent patients after injury, and for some patients, outcomes were favorable after antimicrobial therapy complemented with surgery.

In France, we benefit from the microbiological expertise of university hospital clinical microbiology national network reporting to CNR-MyRMA and from the monthly NTM treatment consilium organized by CNR-MyRMA. The collaborative network probably contributes to the widespread use of macrolides and the avoidance of antimicrobial monotherapy.

Although NTM are known as opportunistic pathogens with infections occurring in patients with underlying conditions or immunodeficiencies (*10*), in our series of EP-MAB, most (62%) patients had no reported immunodeficiency, although a few (15%) had undergone immunologic testing. Underlying inborn errors of immunity in children and adults have been revealed by NTM infections (*25*). We think that among patients with new EP-MAB infections, it is worthwhile to screen for immunodeficiencies (e.g., HIV serology testing or autoantibodies against interferon-γ [*26*]) as first-line tests in association with a clinical examination by a trained physician.

Unlike pulmonary infections, for which exposures are mainly unknown (*10*), in our study, the EP-MAB trigger was identified for 60% of the patients as a penetrating injury associated with healthcare or environmental inoculation. No outbreak was identified. Only the 3 patients with SSTIs remembered an injury, showing that small cutaneous injuries are almost unnoticed, as described previously (*27*–*29*). Considering the literature (*13*,*14*,*30*) and our data, *M. abscessus* infections might result from lack of antiseptic and aseptic procedures before joint infiltration, surgery, and aesthetic procedures including surgical tourism and thus might be avoidable.

Guidelines for treatment of NTM infections have recently been updated but only for pulmonary disease (*10*). Consequently, the recommendations for treatment of extrapulmonary NTM infections are those of the 2007 guidelines (*19*), which proposed a macrolide-based antimicrobial regimen and surgery but had no recommendations for treatment duration. With a cure rate of 80%, our study suggests that EP-MAB may have a much more favorable outcome than pulmonary *M. abscessus* diseases, which could result from the short time to diagnosis, because the median time to diagnosis observed in our study (2 months) aligns with findings from previous studies reporting outbreaks (*13*,*14*,*31*). Such a short time frame could be attributed to clinical similarities with tuberculosis, the ease of accessing sample sites, and the rapid growth ability of *M. abscessus*. It might contribute to the overall better prognosis for EP-MAB than for pulmonary *M. abscessus* infections. The clinical microbiology network reporting to the CNR-MyRMA together with a monthly NTM treatment consilium probably contributes to the use of macrolides and the avoidance of antimicrobial monotherapy. The high proportion of surgeries could be attributed to specific infection sites, primarily involving BJIs and SSTIs. Some EP-MAB resolved spontaneously or with local care or surgery alone, a practice already described for LNIs in children (*32*). Overall, despite the relatively short duration of antimicrobial drug regimens, EP-MAB seems to have a favorable outcome with no sequelae and rare relapses, which contrasts with pulmonary infections, despite longer treatment durations (*10*). The discrepancy underscores the need for prospective studies specific for EP-MAB, specifically the optimal antimicrobial combinations, treatment duration, and criteria for associated or exclusive surgery.

The increase in cases reported to CNR-MyRMA during 2012–2020 was similar to reports from other northern countries (*33*–*36*), which could be attributed to awareness among physicians, along with improvements and standardization of the microbiological diagnosis of NTM (*37*). The increases may also indicate a growing prevalence of *M. abscessus* infections. Our findings underscore the value of increasing knowledge and monitoring of *M. abscessus*. In our cohort, the high number of infections originating from overseas territories of France or from countries in Africa suggests a higher risk for *M. abscessus* infection in tropical regions, consistent with previous reports (*13*,*14*,*31*,*38*). Factors such as climate change, globalization of trade, and medical tourism in tropical regions could contribute to the increased *M. abscessus* infections. Therefore, patients with EP-MAB should be asked about their detailed travel history (*13*). However, we did not identify any outbreaks, and isolates were not genotypically related (data not shown); 30% of the patients had infections diagnosed in metropolitan France, some of them having never traveled abroad. In temperate countries, *M. abscessus* has also been widely reported, primarily affecting patients with pulmonary infections, especially cystic fibrosis (*39*), but the *M. abscessus* environmental reservoir remains largely unknown.

Infection sites were diverse, localized primarily to 3 clinical manifestations: BJIs, SSTIs, and LNIs. Clinical manifestations varied, but classic tuberculosis symptoms (e.g., fever, night sweats, asthenia, or lymph node involvement), were reported for <40% of patients. Conversely, cutaneous signs were observed for 46% of patients, probably linked to a high proportion of SSTIs and BJIs. Enhanced clinical description of skin lesions could help clinicians determine severity and guide treatment decisions, particularly the need for surgery.

One of the main limitations of our study is its retrospective nature. Another limitation is that despite centralization at CNR-MyRMA, EP-MAB cases are not mandatorily reported, resulting in our data not being exhaustive. Less severe cases may not require specific medical management and microbiological diagnosis.

The results from our cohort of EP-MAB enables us to draw the following conclusions: there are no distinct underlying host characteristics but rather an association with penetrating injury and travel to tropical regions, and prognosis is generally favorable when multiple antimicrobial drugs are administered, with surgery if needed. We could not assess differences in outcomes based on clinical localization, causal subspecies, or underlying diseases. Such an assessment would require a collaborative study involving a larger number of cases, categorized by infection sites. While we wait for prospective data to become available, our data may support the benefits of treating EP-MAB with an antimicrobial regimen of >6 months including a macrolide, supplemented with surgery for BJIs and regular patient reassessment.

Appendix Additional information for study of extrapulmonary *Mycobacterium abscessus* infections, France, 2012–2020.
