# Supplementary material for: Extrapulmonary Mycobacterium abscessus Infections, France, 2012–2020
Source: Emerg Infect Dis. 2024 Nov;30(11):2294–302. doi: 10.3201/eid3011.240459 (PMC11521186; doi:10.3201/eid3011.240459)
Supplement: Appendix — Additional information for study of extrapulmonary Mycobacterium abscessus infections, France, 2012–2020. [file 24-0459-Techapp-s1.pdf]

Article DOI: https://doi.org/10.3201/eid3011.240459

EID cannot ensure accessibility for supplementary materials supplied by authors. Readers who have difficulty accessing supplementary content should contact the authors for assistance.

Extrapulmonary *Mycobacterium abscessus* Infections, France, 2012–2020

Appendix

Appendix Table 1. Medical background, geographic origin and symptoms of extrapulmonary *M. abscessus* infections*

N°	Age (years*)	Infection site	Diagnostic sample	Geographic origin	Underlying diseases	B symptoms	Skin involvement	Trauma-infection delay (months)	Health care associated
1	60	BJI (Sp)	Discovertebral biopsy (C6-7)	South America		+	-	24	-
2	80	BJI (Sp)	Discovertebral biopsy (L3-4)	Sub-Saharan Africa		+	-	72	+
3	40	BJI (Art)	Synovial fluid (Sternoclavicular)	Northern Africa		-	-	-	-
4	70	BJI (Art)	Synovial fluid (Knee)	Metropolitan France		+	-	24	+
5	40	BJI (Art)	Synovial fluid (Knee)	French Guyana		+	-	0.25	-
6	60	BJI (Art)	Synovial fluid (Knee)	Metropolitan France		+	+	7	+
7	60	BJI (Art)	Synovial fluid (Knee)	Sub-Saharan Africa		-	-	0.5	+
8	10	BJI (Art)	Synovial fluid (Knee)	Metropolitan France	Explored at NRC, no IEI evidenced	+	-	-	-
9	80	BJI (Art)	Synovial fluid (Wrist)	French West Indies		-	+	3	+
10	70	BJI (PJI)	Synovial fluid (Hip)	Sub-Saharan Africa		-	-	1	+
11	50	BJI (Ost)	Bone (Tibia)	French West Indies		-	+	1	-
12	0	BJI (Ost)	Contiguous abscess (Femur)	Sub-Saharan Africa		-	+	6	+
13	10	BJI (Ost)	Bone (Tibia)	French polynesia		+	-	3	-
14	70	BJI (Ost)	Bone (Heel)	North America	Myasthenia	-	-	6	+
15	100	SSTI	Skin biopsy	Metropolitan France		-	+	-	-
16	50	SSTI	Blood cultures	Metropolitan France	Crohn disease, *M. marinum* SSTI	-	+	-	-
17	60	SSTI	Skin biopsy	Southeast Asia		-	+	72	+
18	30	SSTI	Skin biopsy	Southeast Asia		-	+	1.5	+
19	40	SSTI	Skin biopsy	Metropolitan France		-	+	1	-
20	50	SSTI	Skin biopsy	French West Indies	Kidney allograft	+	+	-	-
21	60	SSTI	Skin biopsy	French West Indies	Kidney allograft, *M. chelonae* SSTI	-	+	-	-
22	80	SSTI	Skin biopsy	French West Indies	Kikuchi & Sjogren Syndromes	-	+	-	-
23	70	SSTI	Skin biopsy	Sub-Saharan Africa	AL amyloidosis, Multiple Myeloma	-	+	-	-
24	80	SSTI	Skin biopsy	Metropolitan France	Idiopathic pulmonary fibrosis	-	+	-	-
25	70	LNI Ing.	Lymph node biopsy	Sub-Saharan Africa		-	+	2	-
26	30	LNI Med.	3 sputums	Central America	Sharp Syndrome	-	-	-	-
27	10	LNI Cerv.	Lymph node biopsy	La Réunion Island	Explored at NRC, no IEI evidenced	-	-	0.25	-
28	40	LNI Cerv.	Lymph node biopsy	Metropolitan France		+	-	-	-
29	80	LNI Med.	2 sputums	New Caledonia		+	-	-	-

N°	Age (years*)	Infection site	Diagnostic sample	Geographic origin	Underlying diseases	B symptoms	Skin involvement	Trauma-infection delay (months)	Health care associated
30	30	LNI Cerv.	Lymph node biopsy	Southeast Asia		-	-	-	-
31	50	LNI Cerv.	Lymph node biopsy	Metropolitan France	Pharyngeal epidermoid carcinoma	-	+	3	+
32	10	LNI Med.	Bronchoalveolar lavage	Sub-Saharan Africa	Steroids for sarcoidosis suspicion	-	-	-	-
33	50	BII	Postoperative abscess	La Réunion Island		-	+	3	+
34	30	BII	Postoperative abscess	La Réunion Island		-	+	1	+
35	40	Breast	Breast abscess	Western Europe		-	+	84	+
36	80	Bacteremia	Blood culture	Eastern Europe	Autograft/lymphoma 10 years ago	+	-	-	-
37	0	IC & B	Blood culture	Middle East	Ewing sarcoma	+	-	1	+
38	70	IC & B	Blood culture	Metropolitan France	Cardia adenocarcinoma	-	-	NA	+
39	50	IC & B	Blood culture	Metropolitan France	Laryngeal epidermoid carcinoma	-	-	NA	+
40	50	MSI	Bronchoalveolar lavage	Sub-Saharan Africa	HIV2, PML	+	-	-	
41	70	MSI	Skin biopsy	La Réunion Island		+	+	1	+
42	20	MSI	Skin biopsy<br>Synovial fluid	French West Indies	Inborn error of Immunity, (NEMO)<br>*M. intracellulare* then *M. avium*<br>SSTI 5 years before	+	+	-	-
43	70	MSI	3 sputums	Sub-Saharan Africa		+	-	-	-
44	60	BTI	Removed biliary prosthesis	Metropolitan France	Kidney allograft	+	-	12	+
45	80	BTI	Surgical site infection after cholecystectomy	Metropolitan France		-	+	3	+

*B, implantable catheter infection & bacteremia; IEI, inborn error of immunity; LNI, lymph nodes infection (cerv, cervical;, med, mediastinal; ing, inguinal), MSI: multisite infection; NA, not available; NRC, national reference centre; PJI, prosthetic joint infection; PML, progressive multifocal leukoencephalopathy; SSTI, skin & soft tissue infection.

Appendix Table 2. Microbiology, treatment and outcome of 45 cases of extrapulmonary *M. abscessus* infections

N°	Age, y*	Infection site	Mab subspecies *erm(41)* sequevar	Initial surgery	Initial intensive antibiotic regimen	Initial antibiotic duration	Maintenance antibiotic regimen	Total antibiotic duration	Outcome
1	60	BJI (Sp)	*Abscessus* T28	-	Clarithromycin, Imipenem, Cefoxitin, Amikacin, Tigecycline	1.5 mo	Clarithromycin, Clofazimine, Linezolid	10 mo	Cured
2	80	BJI (Sp)	*Bolletii*	-	Azithromycin, Cefoxitin, Amikacin, Cotrimoxazole	2 mo	Azithromycin, Imipenem, Linezolid	6 mo	Cured
3	40	BJI (Art)	*Bolletii*	+	Clarithromycin, Imipenem, Amikacin,	2 weeks	Clarithromycin, Linezolid	6 mo	Cured
4	70	BJI (Art)	*Abscessus* T28	+	Clarithromycin, Cefoxitin, Amikacin	1 mo	Clarithromycin, Cefoxitin	5 mo	Cured
5	40	BJI (Art)	*Massiliense*	+	Imipenem, Amikacin	2 weeks	Clarithromycin	6 mo	Cured
6	60	BJI (Art)	*Abscessus* T28	+	Clarithromycin, Cefoxitin, Amikacin	2 mo	Clarithromycin, Cefoxitin	12 mo	Cured
7	60	BJI (Art)	*Abscessus* C28	+	Ofloxacin, Amikacin Rifampin	1 mo	Clarithromycin, Moxifloxacin	2 mo	Cured
8	10	BJI (Art)	*Abscessus* T28	+	Clarithromycin, Imipenem, Amikacin, Tigecycline	1 mo	Clarithromycin, Clofazimine	6 mo	Cured
9	80	BJI (Art)	*Abscessus* T28	+	Azithromycin, Amikacin, Clofazimine	1.5 mo	Azithromycin, Clofazimine	6 mo	Cured
10	70	BJI (PJI)	*Abscessus* T28	+	Clarithromycin, Imipenem Amikacin, Tigecycline, Clofazimine	3 mo	Clarithromycin, Imipenem, Clofazimine	12 mo	Cured
11	50	BJI (Ost)	*Massiliense*	+	Imipenem, Amikacin, Tigecycline, Ciprofloxacin	2 mo	Clarithromycin, Amikacin, Tigecycline	Planned for 12 mo	Relapse then lost to follow-up

N°	Age, y*	Infection site	Mab subspecies *erm(41)* sequevar	Initial surgery	Initial intensive antibiotic regimen	Initial antibiotic duration	Maintenance antibiotic regimen	Total antibiotic duration	Outcome
12	0	BJI (Ost)	*Abscessus* C28	+	Clarithromycin, Amikacin	2 weeks	Clarithromycin	6 mo	Cured
13	10	BJI (Ost)	*Abscessus* T28	+	Surgery alone				Cured
14	70	BJI (Ost)	*Massiliense*	+	Azithromycin, Imipenem, Tigecycline	6 mo	Azithromycin, Imipenem, Tigecycline	6 mo	Cured
15	100	SSTI	*Abscessus* T28	-	Local care alone				Cured
16	50	SSTI	*Abscessus* T28	+	Azithromycin, Amikacin, Linezolid Rifampin				Lost to follow-up at 1 mo
17	60	SSTI	*Abscessus* C28	+	Clarithromycin, Cefoxitin, Amikacin	3 mo	Clarithromycin	9 mo	Cured
18	30	SSTI	*Massiliense*	-	Clarithromycin, Amikacin	1 mo			Cured
19	40	SSTI	*Bolletii*	+	Azithromycin, Imipenem, Amikacin, Tigecycline	2 weeks	Azithromycin, Imipenem, Tigecycline	Planned for 6 mo	Lost to follow-up after 2 weeks
20	50	SSTI	*Massiliense*	-	Azithromycin, Cefoxitin, Amikacin	2 weeks	Azithromycin, Cefoxitin	3 mo	Cured
21	60	SSTI	*Abscessus* T28	-	Clarithromycin, Cefoxitin/Imipenem, Amikacin, Tigecycline, Clofazimine	3 mo	Azithromycin, Clofazimine	15 mo	Cured
22	80	SSTI	*Abscessus* T28	-	Clarithromycin, Cefoxitin	2 mo	Imipenem, Rifabutin, Clofazimine	5 mo	Cured
23	70	SSTI	*Abscessus* T28	-	Clarithromycin, Linezolid Local care	1 week			Cured
24	80	SSTI	*Bolletii*	-	Clarithromycin, Cefoxitin	3 weeks	Clarithromycin	6 mo	Deceased after 6 mo
25	70	LNI Ing.	*Bolletii*	+	Azithromycin,	1.5 mo	Azithromycin,	6 mo	Cured

N°	Age, y*	Infection site	Mab subspecies *erm(41)* sequevar	Initial surgery	Initial intensive antibiotic regimen	Initial antibiotic duration	Maintenance antibiotic regimen	Total antibiotic duration	Outcome
					Imipenem/Cefoxitin, Amikacin, Tigecycline, Clofazimine		Clofazimine		
26	30	LNI Med.	*Bolletii*	-	Spontaneous improvement				Cured
27	10	LNI Cerv.	*Massiliense*	+	Clarithromycin	6 mo			Cured
28	40	LNI Cerv.	*Bolletii*	+	Clarithromycin, Moxifloxacin	1.5 mo	Azithromycin	3 mo	Cured
29	80	LNI Med.	*Abscessus* unspecified	-	Clarithromycin, Cefoxitin, Amikacin	3 weeks	Clarithromycin, Imipenem	1 mo	Lost to follow-up after 1 mo
30	30	LNI Cerv.	*Abscessus* T28	-	Clarithromycin, Linezolid	4 mo	Clarithromycin	6 mo	Cured
31	50	LNI Cerv.	*Massiliense*	+	Surgery alone				Cured
32	10	LNI Med.	*Bolletii*	-	Clarithromycin, Imipenem, Tigecycline	3 mo	Clarithromycin, Inhaled Amikacin, Tigecycline, Linezolid	6 mo	Cured
33	50	BII	*Abscessus* T28	+	Azithromycin, Ciprofloxacin, Cotrimoxazole	2 mo	Cefoxitin, Amikacin, Tigecycline	9 mo	Partial failure of on oral regimen, then cured
34	30	BII	*Abscessus* C28	+	Surgery alone				Cured
35	40	Breast	*Bolletii*	-	Azithromycin, Cefoxitin, Amikacin	6 mo	Azithromycin, Meronem, Amikacin	9 mo	Relapse then cured
36	80	Bacteremia	*Massiliense*	-	Clarithromycin, Imipenem, Amikacin, Ciprofloxacin	1 mo	Clarithromycin, Ciprofloxacin	Long-term	Cured
37	0	IC & B	*Abscessus* T28	+	Clarithromycin,	1 week			Lost to follow-up at 1 week

N°	Age, y*	Infection site	Mab subspecies *erm(41)* sequevar	Initial surgery	Initial intensive antibiotic regimen	Initial antibiotic duration	Maintenance antibiotic regimen	Total antibiotic duration	Outcome
					Cefoxitin, Amikacin				
38	70	IC & B	*Massiliense*	-	Deceased before treatment				Deceased before treatment
39	50	IC & B	*Abscessus* T28	-	Deceased before treatment				Deceased before treatment
40	50	MSI	*Abscessus* C28	-	Deceased before treatment				Deceased before treatment
41	70	MSI	*Abscessus* T28	-	Local care alone				Cured
42	20	MSI	*Bolletii*	+	3 MAB antibiotic treatments in 5 y and multiple surgeries				Relapsed twice, multiple treatment side effects, ultimately improved
43	70	MSI	*Abscessus* T28	+	Azithromycin, Imipenem, Amikacin	1 mo	Azithromycin, Imipenem, Linezolid	3 mo	Cured
44	60	BTI	*Abscessus* C28	+	Clarithromycin, Cefoxitin, Amikacin/Tigecycline	2 weeks	Clarithromycin, Cefoxitin, Clofazimine	3 mo	Cured
45	80	BTI	*Abscessus* T28	-	Spontaneous abscess drainage and local care				Cured

*ROUNDED, BII, breast implant infection; BJI, bone & joint infection; BJI (ART), arthritis; BJI (OST), osteitis; BJI (SP), spondylodiscitis; BTI, biliary tract infection; IC & B, implantable catheter infection and bacteremia; LNI, lymph nodes infection (cerv.: cervical, med.: mediastinal, ing.: inguinal); MSI, multisite infection; PJI, prosthetic joint infection; ssti, skin & soft tissue infection.

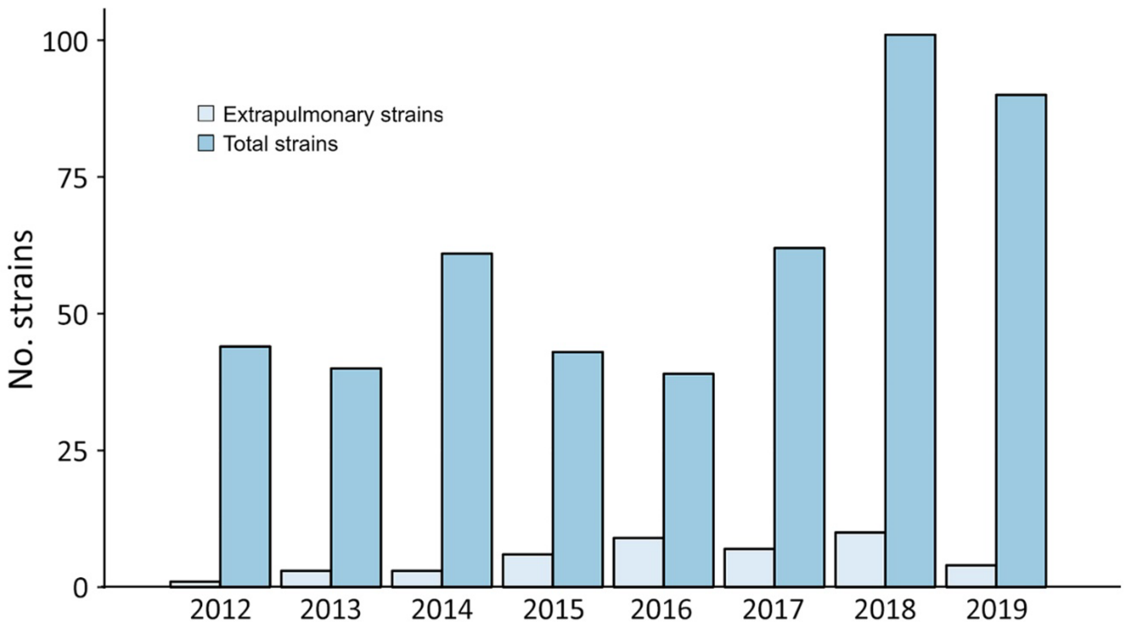

Appendix Figure. Annual count of total & extrapulmonary *M. abscessus* infections strains received by National Reference Centre (France, 2012-2019).
